# Illness Acceptance in Patients with Hidradenitis Suppurativa Depends on Disease Severity and Psychosocial Parameters: An Observational Cross-Sectional Study

**DOI:** 10.3390/jcm15145630

**Published:** 2026-07-17

**Authors:** Marta Szepietowska, Piotr K. Krajewski, Przemyslaw Pacan, Anna Wojas-Pelc, Lukasz Matusiak, Andrzej K. Jaworek

**Affiliations:** 1 Department of Dermatology, Jagiellonian University Medical College, 31-008 Cracow, Poland; anna.wojas-pelc@uj.edu.pl (A.W.-P.); andrzej.jaworek@uj.edu.pl (A.K.J.); 2 Doctoral School of Medical and Health Sciences, Jagiellonian University Medical College, 31-008 Cracow, Poland; 3 Division of Dermatology, Venereology and Clinical Immunology, Faculty of Medicine, Wroclaw University of Science and Technology, 50-370 Wrocław, Poland; piotr.krajewski@pwr.edu.pl (P.K.K.); lukasz.matusiak@pwr.edu.pl (L.M.); 4 Department of Dermato-Venereology, 4th Military Hospital, 50-981 Wrocław, Poland; 5 Department of Psychiatry, Institute of Medical Sciences, Rzeszow University, 35-310 Rzeszow, Poland; ppacan@ur.edu.pl

**Keywords:** hidradenitis suppurativa, illness acceptance, quality of life, psychiatric comorbidities

## Abstract

**Background/Objectives**: Hidradenitis suppurativa (HS) is a chronic inflammatory skin disease associated with substantial physical and psychosocial burden. Illness acceptance is an important component of adaptation to chronic disease, yet it remains underexplored in HS. Therefore, this study aimed to assess illness acceptance in HS patients. **Methods**: This cross-sectional study included 123 consecutive adult HS patients. Illness acceptance was assessed using the Acceptance of Illness Scale (AIS). Disease severity was evaluated using Hurley staging and the International Hidradenitis Suppurativa Severity Score System (IHS4). Pain and itch were measured using the Numeric Rating Scale. Quality of life (QoL) was assessed with the Dermatology Life Quality Index (DLQI) and Hidradenitis Suppurativa Quality of Life (HiSQoL). Depression and anxiety were evaluated using PHQ-9, GAD-7, and HADS. **Results**: The mean AIS score was 31 ± 8.7 points, with 14% of patients showing low and 24% moderate illness acceptance. Lower acceptance was associated with higher Hurley stage (*p* = 0.031). A significant negative correlation between AIS scores and IHS4 values was found (r = −0.26; *p* = 0.013). Additionally, AIS scores correlated negatively with pain (r = −0.22; *p* = 0.019) and itch (r = −0.23; *p* = 0.021). Strong negative correlations were found with QoL impairment (DLQI: r = −0.63; HiSQoL: r = −0.65; *p* < 0.001) and psychological distress, including depression (PHQ-9: r = −0.55; HADS-D: r = −0.60) and anxiety (GAD-7: r = −0.50; HADS-A: r = −0.56; *p* < 0.001 for all). **Conclusions**: Targeting illness acceptance may improve overall patient outcomes.

## 1. Introduction

Hidradenitis suppurativa (HS) is a chronic, recurrent, and inflammatory disorder of the pilosebaceous unit that primarily affects intertriginous areas, including the axillae, groin, and anogenital region. The disease is characterized by painful nodules, abscesses, sinus tract formation, and progressive scarring, leading to substantial physical discomfort and functional impairment [1,2,3]. Despite increasing recognition of HS as a distinct clinical entity, its epidemiology remains incompletely defined. The estimated global prevalence is approximately 1%, although available data vary considerably depending on the methodology used, including differences between self-reported and physician-diagnosed cases [4,5]. Underdiagnosis and misclassification are common, as HS is still insufficiently recognized among both patients and healthcare providers. Importantly, delayed diagnosis has been shown to correlate with increased disease severity and a higher burden of comorbidities at the time of proper recognition [6,7]. HS typically develops in early adulthood and follows a prolonged and relapsing course. In addition to cutaneous manifestations, HS is associated with numerous systemic comorbidities, such as metabolic syndrome, cardiovascular disease, inflammatory bowel disease, and spondyloarthropathies [2,8]. Importantly, the burden of HS extends far beyond physical symptoms, profoundly affecting patients’ quality of life (QoL), social functioning, intimate relationships, and occupational performance [9,10]. Previous studies have demonstrated that HS is linked with significant psychological morbidity, including depression and anxiety, highlighting its multidimensional impact and the need for comprehensive, patient-centered management [11].

The pathogenesis of HS is complex and multifactorial, involving an interplay between genetic predisposition, environmental factors, and dysregulated immune responses. Current evidence suggests that follicular occlusion and subsequent rupture of the pilosebaceous unit represent initiating events that activate innate and adaptive immune pathways. Inflammatory mediators, including tumor necrosis factor (TNF), interleukin (IL)-1β, IL-17, and other cytokines, play a central role in sustaining chronic inflammation and tissue destruction [1,2,3]. Moreover, transcriptomic studies have demonstrated significant alterations in gene expression profiles within lesional and perilesional skin, particularly involving pathways related to inflammation and extracellular matrix remodeling [12]. These mechanisms contribute to the formation of sinus tracts, fibrosis, and irreversible scarring characteristic of advanced disease. In addition to biological factors, lifestyle and environmental influences such as smoking, obesity, and mechanical friction are recognized contributors to HS development and progression [1,2,3].

Importantly, HS remains a relatively underrecognized condition not only in clinical practice but also in public awareness [13]. Despite its significant impact on patients’ daily functioning and well-being, the disease is often stigmatized and associated with feelings of shame and social isolation [14,15]. This lack of awareness, combined with the intimate localization of lesions and chronic relapsing course, may contribute to delays in seeking medical care and difficulties in coping with the disease. Consequently, HS represents not only a dermatological disorder but also a complex biopsychosocial condition requiring a multidimensional approach to patient care [2].

Acceptance of illness is a key psychological construct reflecting the degree to which an individual adapts to the limitations imposed by a chronic condition. Higher levels of illness acceptance are associated with better emotional adjustment, reduced psychological distress, and improved overall well-being. Conversely, low acceptance may lead to maladaptive coping strategies, increased levels of anxiety and depression, and poorer QoL [16,17]. In chronic dermatological diseases, such as psoriasis or acne, illness acceptance has been shown to correlate with disease severity, symptom burden, stigmatization, and QoL impairment [18,19,20]. Furthermore, studies conducted in other chronic conditions indicate that illness acceptance is closely linked with broader psychosocial parameters, including hopelessness, health perception, and functional independence [21,22]. Despite growing recognition of its importance, illness acceptance remains relatively underexplored in HS, particularly in the context of its complex psychosocial burden.

Therefore, the aim of the present study was to assess the level of illness acceptance in patients with HS and to investigate its associations with a wide range of clinical and psychosocial parameters. Specifically, we sought to examine the relationships between illness acceptance and disease severity, subjective symptoms (such as pain and itch), QoL, and psychological factors, including depression and anxiety. By providing a comprehensive evaluation of illness acceptance in HS, this study aims to contribute to a better understanding of psychological adaptation in this population and to identify potential targets for holistic therapeutic interventions.

## 2. Materials and Methods

### 2.1. Study Design and Participants

This cross-sectional observational study was conducted among consecutive adult outpatients diagnosed with HS, who attended routine dermatology consultations at tertiary referral centers in Wroclaw and Cracow, Poland. The diagnosis of HS was established by experienced dermatologists based on well-established clinical criteria. Inclusion criteria comprised the following: (1) age ≥ 18 years; (2) a confirmed diagnosis of HS; (3) attendance at one of the participating outpatient dermatology clinics during the study period; (4) ability to understand and independently complete the study questionnaires; and (5) provision of written informed consent. Exclusion criteria included the following: (1) age below 18 years; (2) refusal to participate; (3) inability to complete the questionnaires because of cognitive or language difficulties; and (4) incomplete questionnaire data. A total of 132 consecutive patients were invited to participate, of whom 123 completed all required assessments and were included in the final analysis (response rate: 93%). Patients were asked to complete a set of validated questionnaires assessing illness acceptance, psychological status, and QoL. All instruments were provided in Polish language versions. Demographic and clinical data were collected using a structured questionnaire and are presented in Table 1.

The study protocol was approved by the local ethics committee (Ethics Committee of Lower Silesian Medical Chamber, Wroclaw, Poland—decision number 12/BNBP/2025), and the study was conducted in accordance with the Declaration of Helsinki.

### 2.2. Clinical Severity and Symptom Assessment

Disease severity was assessed using two validated instruments: the Hurley staging system and the International Hidradenitis Suppurativa Severity Score System (IHS4). The Hurley classification categorizes HS into three stages based on the extent of inflammatory lesions and scarring [23], whereas IHS4 provides a dynamic numerical assessment of disease activity based on the number of nodules, abscesses, and draining tunnels. As measured by the IHS4 total score, HS severity is categorized as mild (up to 3 points), moderate (4–10 points), or severe (greater than 10 points) [24].

Subjective symptoms were assessed using the Numeric Rating Scale (NRS). Patients were asked to rate the intensity of pain and itch on an 11-point scale ranging from 0 (no symptoms) to 10 (worst imaginable symptoms) [25,26,27]. The overall intensity of the worst symptoms throughout the whole course of the disease was considered.

### 2.3. Illness Acceptance Assessment

Illness acceptance was assessed using the Acceptance of Illness Scale (AIS), originally developed by Felton, Revenson, and Hinrichsen, a widely used and validated self-report instrument designed to evaluate psychological adaptation to chronic disease [28,29]. The AIS consists of 8 statements describing the negative consequences of ill health, such as limitations imposed by the disease, reduced independence, and decreased self-esteem. Each item is rated on a 5-point Likert scale ranging from 1 (“strongly agree”) to 5 (“strongly disagree”), resulting in a total score between 8 and 40 points [28].

Higher AIS scores indicate better illness acceptance and more effective adaptation to the disease, whereas lower scores reflect poor acceptance and greater psychological discomfort. To facilitate interpretation of the results, illness acceptance levels were categorized based on established cut-off values for the AIS. Scores ranging from 8 to 18 points indicate low illness acceptance, reflecting poor psychological adaptation and significant discomfort related to the disease. Scores between 19 and 29 indicate a moderate level of acceptance, suggesting partial adaptation to illness-related limitations. Scores of 30 points or higher are considered indicative of high illness acceptance, representing good adjustment and effective coping with the disease. This categorization has been widely used in studies assessing illness acceptance in chronic conditions, including dermatological diseases [28,29].

### 2.4. Quality of Life Assessment

QoL was evaluated using dermatology-specific instruments: the Dermatology Life Quality Index (DLQI) and the Hidradenitis Suppurativa Quality of Life (HiSQoL) questionnaire. The DLQI consists of 10 items assessing the impact of skin disease on daily functioning over the previous week, with a total score ranging from 0 to 30 points. Higher scores indicate greater impairment of QoL [30]. The HiSQoL is a disease-specific instrument developed for HS patients that captures multiple domains of disease burden, including physical symptoms, emotional impact, and social functioning. The higher the scores, the more impaired QoL [31,32].

### 2.5. Psychological (Depression and Anxiety) Assessments

Depressive symptoms were assessed using the Patient Health Questionnaire-9 (PHQ-9) and the depression subscale of the Hospital Anxiety and Depression Scale (HADS-D). The PHQ-9 is a 9-item self-administered questionnaire evaluating the frequency of depressive symptoms over the past two weeks, with total scores ranging from 0 to 27 points. Higher scores indicate greater severity of depression [33,34]. The HADS-D subscale consists of 7 items scored from 0 to 3 points, with a maximum score of 21 points. Similarly to the previous scale, higher scores indicate greater severity of depressive symptoms [35].

Anxiety was assessed using the Generalized Anxiety Disorder-7 (GAD-7) questionnaire and the anxiety subscale of the Hospital Anxiety and Depression Scale (HADS-A). The GAD-7 consists of 7 items rated on a 4-point Likert scale (0–3), with total scores ranging from 0 to 21 points [36]. The HADS-A subscale includes 7 items, scored 0–3 points with a maximal score of 21 points. Higher scores reflect greater anxiety severity [35].

The use of several instruments to evaluate depression and anxiety was deliberate, aiming to capture different yet complementary aspects of psychological distress. The PHQ-9 and GAD-7, which are based on DSM criteria, enable a quantitative measurement of symptom severity, whereas the HADS was specifically developed for patients with somatic conditions and reduces the impact of physical symptoms on the assessment [33,34,35,36,37]. This strategy allowed for a more thorough evaluation of the psychological burden in patients with HS and was used previously by our group in other studies on HS [11].

### 2.6. Statistical Analysis

Statistical analysis was conducted using IBM SPSS Statistics version 26 (SPSS Inc., Chicago, IL, USA). Continuous variables were presented as mean ± standard deviation (SD) or median with interquartile range (IQR), depending on data distribution. Categorical variables were expressed as absolute numbers and percentages. The normality of data distribution was assessed using the Shapiro–Wilk test. Depending on distribution, differences between groups were analyzed using Student’s *t*-test or the Mann–Whitney U test for two groups, and one-way ANOVA or the Kruskal–Wallis test for multiple groups. Correlations between variables were evaluated using Pearson’s or Spearman’s correlation coefficients, as appropriate. A *p*-value < 0.05 was considered statistically significant. 

## 3. Results

### 3.1. Illness Acceptance and Its Items

The overall distribution of illness acceptance levels showed that 14% of HS patients presented with low illness acceptance, while nearly one quarter demonstrated a moderate level of acceptance (24%). The majority of the study group exhibited high illness acceptance (62%). This distribution was similar across gender subgroups, although a slightly higher proportion of low acceptance was observed among females (16%) compared to males (12%) (Table 2).

The detailed analysis of individual items of the AIS is presented in Table 3. Mean scores for individual items ranged from 3.3 ± 1.5 to 4.3 ± 1.2 points. The lowest mean scores were observed for statements related to difficulties in adapting to disease-related limitations (3.3 ± 1.5 points) and inability to engage in preferred activities (3.3 ± 1.5 points), suggesting that these aspects constituted the most challenging areas for patients. In contrast, the highest levels of acceptance were noted for items reflecting self-worth (4.3 ± 1.1 points), perception of being a burden to others (4.2 ± 1.2 points), and anticipated loss of independence (4.1 ± 1.3 points), indicating that these concerns were less frequently endorsed (Table 3).

**Figure 1:**
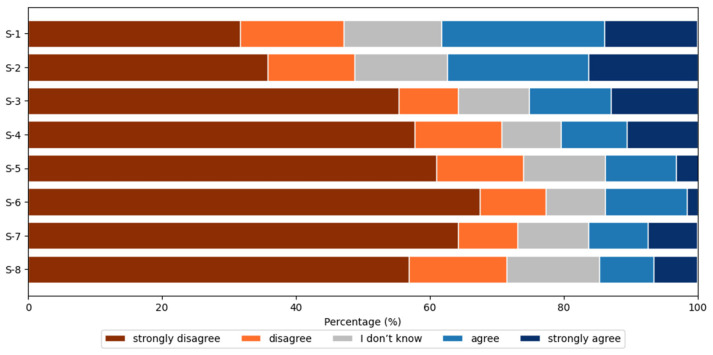
Distribution of patients’ answers to the Acceptance of Illness Scale (AIS) statements.

The above findings reflect comparatively higher proportions of agreement noted for statements addressing functional limitations and daily activity restrictions. Approximately 38% of patients agreed or strongly agreed that they experienced difficulties adapting to the limitations imposed by the disease (S-1), and 37% reported that their condition prevented them from doing what they enjoy most (S-2) (Table 4, Figure 1).

### 3.2. Illness Acceptance and Clinical Disease Severity

**Figure 2:**
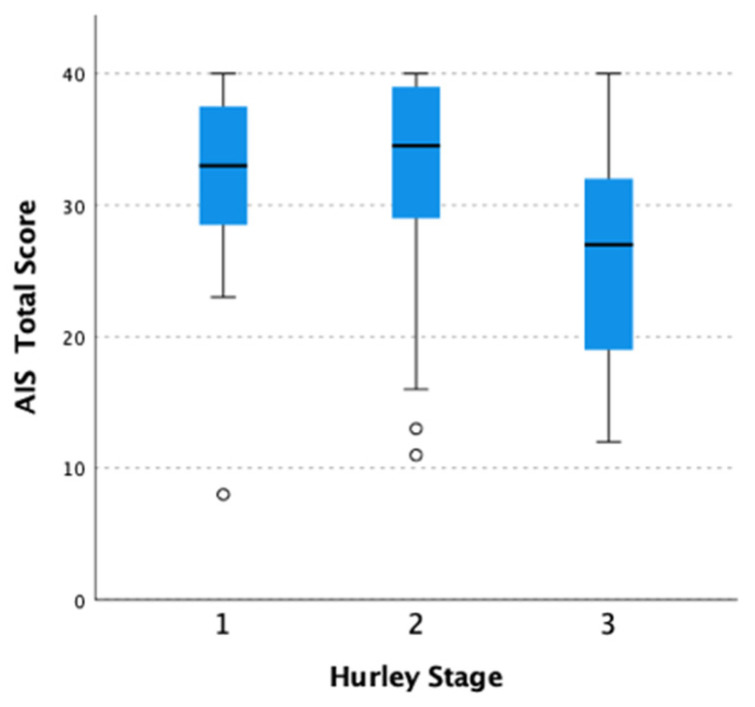
Illness acceptance according to Hurley’s stages.

A statistically significant association between illness acceptance and clinical severity of HS was observed when disease severity was assessed using the Hurley staging system (Figure 2).

**Figure 3:**
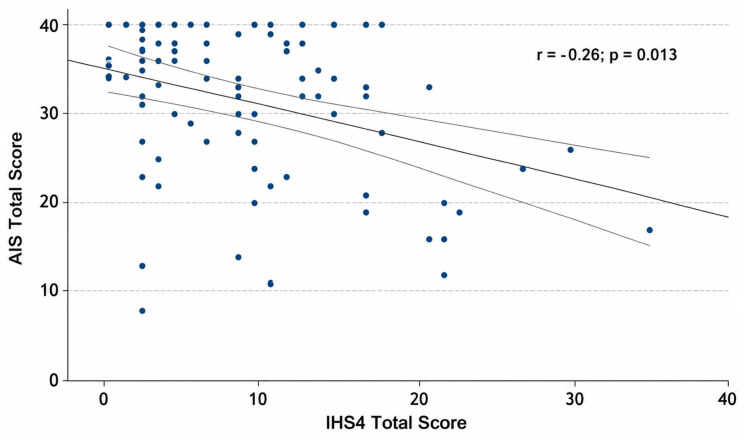
Correlation between the Acceptance of Illness Scale (AIS) and the International Hidradenitis Suppurativa Severity Score System (HIS-4).

Patients with more advanced disease (Hurley stage III) demonstrated markedly lower levels of illness acceptance (mean AIS: 26 ± 8.5 points) compared to those with Hurley stage I (32 ± 8.4 points) and stage II (33 ± 7.9 points). The overall difference between groups was statistically significant (*p* = 0.031). Post hoc analysis revealed a significant difference between patients with Hurley stage III and stage II disease (*p* = 0.025), whereas no significant differences were observed between stages I and II. In contrast, when disease severity was evaluated using the IHS4, no significant differences in AIS scores were found between severity categories. Nevertheless, a gradual decrease in illness acceptance was observed with increasing disease severity, with mean AIS scores of 33 ± 8.3 points in mild disease, 32 ± 7.6 points in moderate disease, and 30 ± 8.9 points in severe disease. Further analysis demonstrated a weak but statistically significant negative correlation between AIS scores and IHS4 values (r = −0.26; *p* = 0.013), indicating that higher disease activity was associated with lower illness acceptance (Figure 3).

**Figure 4:**
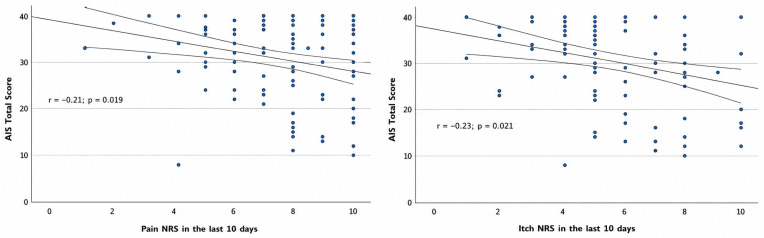
Correlations between the Acceptance of Illness Scale (AIS) and pain and itch intensity (NRS—Numerical Rating Scale).

Illness acceptance was also significantly associated with subjective symptom burden. A weak negative correlation was found between AIS scores and pain intensity (r = −0.21; *p* = 0.019), as well as between AIS scores and itch severity (r = −0.23; *p* = 0.021) (Figure 4).

### 3.3. Illness Acceptance and Psychosocial Aspects

**Figure 5:**
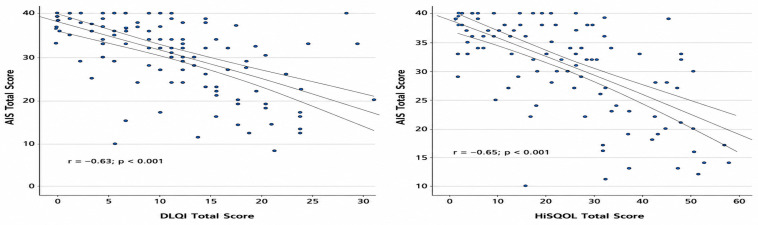
Correlations between the Acceptance of Illness Scale (AIS) and quality of life measurements (DLQI—Dermatology Life Quality Index; HiSQoL—Hidradenitis Suppurativa Quality of Life).

A strong and statistically significant relationship was observed between illness acceptance and QoL impairment. AIS scores demonstrated a pronounced negative correlation with both dermatology-specific and disease-specific QoL measures. In particular, higher levels of illness acceptance were associated with lower impairment in the DLQI (r = −0.63; *p* < 0.001) and the HiSQoL questionnaire (r = −0.65; *p* < 0.001) (Figure 5).

**Figure 6:**
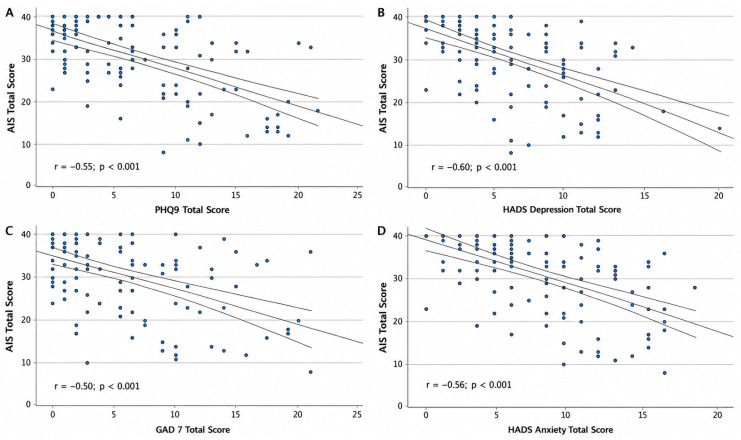
Correlations between the Acceptance of Illness Scale (AIS) and measures for depression ((**A**) PHQ-9—Patient Health Questionnaire-9; (**B**) HADS—Hospital Anxiety and Depression Scale—Depression) and anxiety ((**C**) GAD-7—Generalized Anxiety Disorder-7; (**D**) Hospital Anxiety and Depression Scale—Anxiety).

Additionally, illness acceptance was strongly associated with the severity of depressive symptoms. AIS scores demonstrated a strong, significant negative correlation with all applied measures of depression, including the PHQ-9 (r = −0.55; *p* < 0.001) and the depression subscale of the Hospital Anxiety and Depression Scale (HADS-D) (r = −0.60; *p* < 0.001). (Figure 6A,B). A similar pattern was observed for anxiety. AIS scores were significantly negatively correlated with both the GAD-7 scale (r = −0.50; *p* < 0.001) and the anxiety subscale of the Hospital Anxiety and Depression Scale (HADS-A) (r = −0.56; *p* < 0.001) (Figure 6C,D).

## 4. Discussion

Illness acceptance is a key psychological construct reflecting an individual’s ability to adapt to the limitations imposed by chronic disease. It encompasses cognitive, emotional, and behavioral components, including the recognition of disease-related constraints, adjustment of expectations, and maintenance of self-esteem and autonomy [16,17]. Higher levels of illness acceptance have been consistently associated with better psychological well-being, improved coping strategies, and enhanced QoL, whereas low acceptance is linked with emotional distress, maladaptive coping, and reduced treatment adherence [38,39,40]. Therefore, illness acceptance is increasingly recognized as an important target for holistic, patient-centered care in chronic diseases. From a broader perspective, illness acceptance should be understood as a dynamic and multidimensional process rather than a fixed state. It involves not only cognitive acknowledgment of the disease but also emotional adjustment and behavioral adaptation to its consequences. Importantly, it does not imply the absence of negative emotions but rather the ability to integrate the experience of illness into one’s life narrative while maintaining a sense of identity and purpose. Moreover, acceptance is not linear. It fluctuates over time, particularly in response to disease exacerbations or changes in life circumstances [22,41].

Despite the growing interest in psychodermatology, illness acceptance remains insufficiently explored in HS. While numerous studies have addressed QoL impairment, depression, and anxiety in HS [10,11,42,43], the concept of illness acceptance has received relatively little attention. This is particularly noteworthy given the unique clinical characteristics of HS, including chronic pain, recurrent inflammation, scarring, and localization in intimate body areas, all of which may profoundly affect psychological adaptation [2,3]. To the best of our knowledge, this study represents the most comprehensive analysis of illness acceptance in HS, examining its associations with both clinical severity and a broad spectrum of psychosocial parameters.

When comparing our findings with other dermatological conditions, several important similarities emerge. In psoriasis, illness acceptance has been shown to be moderate overall, with significant variability among patients. Similarly, Jankowiak et al. [19] reported moderate levels of acceptance (mean AIS ~24 points), with a clear relationship between illness acceptance and QoL impairment. In contrast, studies in acne patients, particularly younger populations, have demonstrated considerably higher levels of illness acceptance, with the majority of patients presenting high acceptance levels [20]. These differences may be explained by variations in disease severity, visibility, chronicity, symptom burden, and perceived stigma. Compared with psoriasis, our cohort of HS patients appeared to be associated with relatively higher levels of illness acceptance, which may reflect adaptation over time despite the substantial burden of the disease, although direct comparisons should be interpreted with caution due to methodological differences between studies. Moreover, one should consider that our HS patients were recruited in specialized HS centers, where patients usually experience a more holistic approach to their disease. This also might have a potential influence on higher illness acceptance levels.

Our findings on illness acceptance also align with data from other chronic conditions, where illness acceptance has been shown to play a central role in psychological adaptation [21,44,45]. For example, in patients with stroke, higher illness acceptance is associated with better health perception and lower levels of hopelessness [21]. In patients with cardiovascular diseases, chronic obstructive pulmonary disease, and multiple sclerosis, illness acceptance has been shown to be influenced by both clinical and psychosocial factors, including disease activity, remission status, and the presence of depressive symptoms [22]. Notably, higher acceptance levels are typically observed in patients during periods of remission, whereas exacerbations and symptom progression are associated with decreased acceptance [22]. Furthermore, depressive symptoms have been identified as one of the strongest negative predictors of illness acceptance, with a clear inverse relationship between depression severity and acceptance levels [18,20]. These observations support the notion that illness acceptance is a universal construct across chronic diseases, influencing both emotional well-being and functional outcomes. They also reinforce the concept that illness acceptance is a transdiagnostic construct shaped by both disease-related and psychological factors.

An important finding of the present study is the significant association between illness acceptance and disease severity. Patients with more advanced HS (Hurley stage III) demonstrated significantly lower AIS scores compared to those with milder disease. This is consistent with findings in psoriasis and acne, where greater disease severity has been associated with lower illness acceptance [18,20]. Severe disease is typically accompanied by more pronounced physical limitations, increased symptom burden, and greater social stigma, all of which may impair psychological adaptation. Interestingly, while no significant differences were observed between IHS4 severity categories, a significant negative correlation between IHS4 and AIS scores was identified. This suggests that dynamic measures of disease activity may capture more subtle relationships with illness acceptance than categorical classifications. Subjective symptoms, particularly pain and itch, also played a significant role in illness acceptance. Both symptoms were negatively correlated with AIS scores, indicating that higher symptom burden is associated with poorer psychological adaptation. This finding is in line with previous research on psoriasis, where itch intensity has been identified as a key factor influencing illness acceptance [18]. In HS, pain is a predominant and often debilitating symptom, which may further exacerbate emotional distress and hinder adaptation to the disease [46]. These results highlight the importance of effective symptom control not only for physical relief but also for improving psychological outcomes.

One of the most prominent findings of this study is the strong association between illness acceptance and QoL. AIS scores demonstrated robust negative correlations with both DLQI and HiSQoL, indicating that patients with higher acceptance experience substantially lower QoL impairment. These findings are consistent with previous studies in dermatological conditions, where illness acceptance has been identified as a key determinant of QoL [18,19,20]. Notably, the slightly stronger correlation observed for the HS-specific HiSQoL questionnaire suggests that illness acceptance may be particularly relevant in capturing disease-specific aspects of burden.

Equally important is the strong relationship between illness acceptance and psychological distress. In our study, AIS scores were strongly negatively correlated with both depressive and anxiety symptoms across multiple validated instruments. These findings are consistent with broader literature indicating that lower illness acceptance is associated with higher levels of depression, anxiety, and psychological maladaptation. In dermatological populations, reduced illness acceptance has also been linked to body image disturbances and increased risk of psychiatric comorbidities [18,19,20,47,48]. Given the high prevalence of mental health disorders in HS, these results underscore the importance of integrating psychological assessment and support into routine clinical care.

Several limitations of this study should be acknowledged. First, due to its cross-sectional design, causal relationships between illness acceptance and clinical or psychosocial variables cannot be established. The observed associations should therefore be interpreted as correlational rather than indicative of directionality. Second, the study was conducted in a relatively limited sample recruited from tertiary dermatology centers, which may restrict the generalizability of the findings. Patients treated in specialized centers often present with more severe or long-standing disease, potentially introducing selection bias. Moreover, in specialized centers, their expectations of receiving high-quality service might be higher, which could also influence the presented results. Third, all psychological and symptom-related data were based on self-reported questionnaires. Although validated instruments were used, this approach may be subject to reporting bias, including underestimation or overestimation of symptoms such as depression and anxiety. Another limitation is the lack of longitudinal assessment. Illness acceptance is a dynamic process that may change over time depending on disease course, treatment response, and life circumstances. A longitudinal design would provide a more comprehensive understanding of these changes. Moreover, although multiple validated tools were used to assess psychological burden, potential confounding factors such as coping strategies, social support, personality traits, or previous psychiatric treatment were not evaluated, which may have influenced the observed relationships. Finally, the present study was primarily designed to explore associations between illness acceptance and a broad range of clinical and psychosocial variables using bivariate analyses. Although this approach allowed us to comprehensively characterize these relationships, it does not permit identification of independent predictors of illness acceptance after adjustment for potential confounders. Future studies based on larger cohorts and prospective designs should incorporate multivariable regression models to determine which clinical and psychological variables independently contribute to illness acceptance in HS.

## 5. Conclusions

Illness acceptance is closely associated with disease severity, QoL, and psychological distress in patients with HS. These findings suggest that illness acceptance may represent a clinically relevant psychosocial construct. Interventions targeting illness acceptance warrant further investigation in prospective longitudinal studies. From a clinical perspective, our results emphasize the need for a holistic approach to HS management, incorporating not only pharmacological treatment but also psychosocial interventions aimed at improving illness acceptance. Patient education, cognitive-behavioral therapy, and supportive counseling may enhance coping strategies and ultimately improve patient outcomes.

## Figures and Tables

**Table 1:** Clinical characteristics of the hidradenitis suppurativa study population.

Variable	Total	Females	Males	*p*-Value
Number of patients, n (%)	123	62 (50.4%)	61 (49.6%)	
Age (years), mean ± SD	36 ± 12.4	36 ± 13.0	36 ± 11.9	NS
Disease duration (years), mean ± SD	10 ± 6.4	10 ± 7.2	10 ± 5.6	NS
Hurley stage, n (%)				0.003
Stage I	19 (15.4)	11 (17.7)	8 (13.1)	
Stage II	86 (69.9)	49 (79.0)	37 (60.7)	
Stage III	18 (14.7)	2 (3.2)	16 (26.2)	
HIS-4, mean ± SD	9 ± 7.0	8 ± 6.6	10 ± 7.3	NS
HIS-4 categories, n (%)				NS
IHS-4 ≤ 3 points, mild	35 (28.4%)	23 (37.1%)	12 (19.7%)	
IHS-4 4–10 points, moderate	43 (35%)	22 (35.5%)	21 (34.4%)	
IHS-4 > 10 points, severe	45 (36.6%)	17 (27.4%)	28 (45.9%)	
Itch (NRS points), mean ± SD	5.7 ± 2.2	5.9 ± 2.3	5.5 ± 2.1	NS
Pain (NRS points), mean ± SD	7.3 ± 2.1	7.8 ± 2.1	6.8 ± 2.1	NS

n—number; SD—standard deviation; IHS-4—International Hidradenitis Suppurativa Severity Score System; NRS—Numeral Rating Scale; NS—non-significant.

**Table 2:** The degree of illness acceptance of the studied hidradenitis suppurativa patients.

Illness Acceptance	Whole Group n, (%)	Males n, (%)	Females n, (%)	*p*
Low	17 (14)	7 (12)	10 (16)	NS
Moderate	30 (24)	15 (25)	15 (24)	NS
High	76 (62)	39 (63)	37 (60)	NS

n—number; NS—non-significant.

**Table 3:** Illness acceptance measured among hidradenitis suppurativa patients using the Acceptance of Illness Scale (AIS).

Item of AIS	Mean ± SD [Points]
1. I have difficulty adapting to the limitations imposed by the disease.	3.3 ± 1.5
2. I cannot do what I like best because of my health condition.	3.3 ± 1.5
3. My illness makes me sometimes feel unwanted.	3.8 ± 1.5
4. Health problems make me more dependent on others than I would prefer.	4 ± 1.4
5. My illness makes me a burden for my family and friends.	4.2 ± 1.2
6. Because of my health condition, I do not feel like a truly valuable person.	4.3 ± 1.1
7. I will never be as self-sufficient to the extent to which I would like to be.	4.1 ± 1.3
8. I think people around me often feel embarrassed because of my illness.	4 ± 1.3

AIS—Acceptance of Illness Scale.

**Table 4:** Distribution of responses on the Acceptance of Illness Scale (AIS).

	S-1	S-2	S-3	S-4	S-5	S-6	S-7	S-8
Strongly agree	14%	16%	13%	11%	3%	2%	7%	7%
Agree	24%	21%	12%	10%	11%	12%	9%	8%
I do not know	15%	14%	11%	9%	12%	9%	11%	14%
Disagree	15%	13%	9%	13%	13%	10%	9%	14%
Strongly disagree	32%	36%	55%	57%	61%	67%	64%	57%

S—Statement according to the Acceptance of Illness Scale (AIS).

## Data Availability

Data are available upon reasonable request from the corresponding authors.

## Abbreviations

The following abbreviations are used in this manuscript:

Abbreviation	Definition
AIS	Acceptance of Illness Scale
DLQI	Dermatology Life Quality Index
GAD-7	Generalized Anxiety Disorder-7
HADS	Hospital Anxiety and Depression Scale
HADS-A	Hospital Anxiety and Depression Scale—Anxiety
HADS-D	Hospital Anxiety and Depression Scale—Depression
HiSQoL	Hidradenitis Suppurativa Quality of Life
HS	Hidradenitis Suppurativa
IHS4	International Hidradenitis Suppurativa Severity Score System
NRS	Numerical Rating Scale
PHQ-9	Patient Health Questionnaire-9
QoL	Quality of Life
SD	Standard Deviation
